# Locally-deployed vs. cloud-based AI in healthcare: evaluating DeepSeek-R1:8b, DeepSeek-R1, and ChatGPT o3-mini-high for complex medical diagnostics

**DOI:** 10.3389/fdgth.2026.1785443

**Published:** 2026-04-22

**Authors:** Ning He, Lin Yang, Xinhong Hu, Yuanfang He

**Affiliations:** 1 Department of Urology, Hanzhong Central Hospital, Hanzhong, Shaanxi, China; 2 Department of Radiology, Hanzhong Central Hospital, Hanzhong, Shaanxi, China; 3 Department of Clinical Laboratory, Hanzhong Central Hospital, Hanzhong, Shaanxi, China

**Keywords:** clinical decision support systems, DeepSeek, edge AI, knowledge-based systems, large language models, local AI deployment, medical diagnosis, model reliability

## Abstract

Reasoning large language models are increasingly considered for healthcare-related artificial intelligence applications, but their practical value depends not only on diagnostic accuracy, but also on responsiveness and operational reliability. In this study, we benchmarked six model settings on 1,000 questions from the MedQA dataset: DeepSeek-R1, its distilled 8-billion-parameter local variant DeepSeek-R1:8b, ChatGPT o3-mini-high, and their knowledge-base–augmented counterparts. We evaluated performance across three dimensions: diagnostic accuracy, response latency, and first-attempt connection reliability. DeepSeek-R1 achieved the highest accuracy (89.5%, 95% CI: 87.4–91.2) but showed substantially longer response times (median 26.54 s) and higher connection failure rates (4.6%). ChatGPT o3-mini-high responded faster (median 10.05 s) and showed the most favorable tail-latency profile, but its accuracy (78.2%, 95% CI: 75.5–80.7) was lower than that of DeepSeek-R1. The locally deployed DeepSeek-R1:8b demonstrated markedly stronger connection reliability (failure rate 0.2%, 95% CI: 0.0%–0.5%) but substantially reduced accuracy (55.0%, 95% CI: 51.9%–58.5%). Knowledge-base augmentation did not consistently improve performance; for DeepSeek-R1, it significantly reduced accuracy by 4.36% (p=0.0002), while no significant benefit was observed for the other models. These findings show that reasoning model performance in medical question answering is best understood as a trade-off among accuracy, latency, connection reliability, and deployment mode, and that retrieval augmentation is not universally beneficial. More broadly, this study provides deployment-relevant benchmarking evidence for evaluating reasoning models in healthcare-related settings, while also indicating the need for richer knowledge resources and more realistic task environments before such systems can be meaningfully assessed for real-world clinical use.

## 1 Introduction

Large language models (LLMs) have rapidly evolved from general-purpose text generators into systems capable of solving increasingly complex medical question-answering tasks. Early studies showed that ChatGPT could achieve passing or near-passing performance on the United States Medical Licensing Examination (USMLE), suggesting their potential utility in medical education and structured knowledge retrieval [1]. Subsequent work has further validated the strong performance of more advanced models on challenging medical reasoning tasks. For instance, GPT-4 demonstrated strong capability on medical challenge problems [2], while Med-PaLM 2 achieved expert-level performance on benchmarks such as MedQA, underscoring the growing potential of LLMs for high-level medical question answering [3].

At the same time, recent studies have emphasized that benchmark accuracy alone is insufficient for characterizing the quality of medical reasoning. Chen et al. demonstrated that model performance can vary substantially on more difficult medical question-answering datasets that also require explanation quality, suggesting that a model’s apparent strength depends not only on answer correctness but also on benchmark difficulty and reasoning transparency [4]. This consideration is particularly pertinent to the current generation of reasoning models. These models are designed to allocate additional computational resources to multi-step inference and, consequently, may exhibit different behaviors from conventional LLMs in medical decision-support scenarios.

A particularly important recent development is the emergence of open-weight reasoning models, such as DeepSeek-R1 [5]. DeepSeek has attracted considerable international attention due to its strong reasoning capabilities, open model ecosystem, and potential to broaden access to advanced AI systems [6, 7]. In healthcare, these attributes are especially pertinent, as open and locally deployable models may offer practical advantages over cloud-only, proprietary systems. Local deployment is increasingly recognized as a critical direction for both research and implementation, enabling institutions to retain control over sensitive data, reduce dependence on external connectivity, and tailor models to local infrastructure and workflow constraints. Within this context, DeepSeek’s open-weight architecture and its smaller, deployable variants render it a particularly compelling candidate for investigating the real-world trade-offs between reasoning performance and operational feasibility. In parallel, comparative studies have suggested that advanced reasoning models, including ChatGPT o1 and DeepSeek-R1, may exhibit meaningful differences in clinical decision-support behavior, further motivating direct benchmarking of this model class in medicine [8].

In parallel with the development of reasoning models, retrieval-augmented generation (RAG) has also become an important direction in medical LLM research. Prior work has demonstrated that carefully designed medical RAG pipelines can enhance factual grounding and overall performance [9]. More recently, studies on rationale-guided retrieval have demonstrated that RAG effectiveness is highly contingent on retrieval strategy, corpus composition, and context filtering, rather than being uniformly beneficial [10]. Collectively, these findings suggest that knowledge-base augmentation should not be assumed to confer automatic performance gains, particularly when applied to reasoning models.

Despite rapid progress, several important gaps persist in the literature. First, most prior evaluations of medical LLMs have focused predominantly on accuracy, with considerably less attention paid to deployment-oriented metrics such as response latency, tail latency, and connection reliability. Second, although reasoning models are becoming increasingly prominent, relatively few studies have specifically examined their performance as a distinct model category in medical question-answering tasks. Third, direct comparisons between cloud-based and locally deployed reasoning models remain limited, despite the critical relevance of this distinction for healthcare environments characterized by varying privacy requirements, computational resources, and network conditions. Finally, while RAG has shown promise in medical QA, its practical implications for latency, stability, and overall deployment trade-offs have yet to be systematically characterized under standardized benchmarking conditions.

To address these gaps, we evaluated three reasoning LLM configurations—DeepSeek-R1, DeepSeek-R1:8b, and ChatGPT o3-mini-high—alongside their knowledge-base-augmented variants on 1,000 questions from the MedQA dataset. Rather than focusing solely on benchmark accuracy, we assessed a broader performance profile including response time, tail latency, and first-attempt connection failures. By systematically comparing cloud-based and locally deployed models, as well as RAG-enhanced and non-RAG configurations, this study aims to elucidate the trade-offs between reasoning capability, responsiveness, and operational reliability. In doing so, our work extends prior medical QA benchmarking from a capability-centered perspective toward a more deployment-aware evaluation framework.

## 2 Materials and methods

The following section provides an overview of the tools and methodologies used for local deployment of the DeepSeek-R1:8b model, knowledge base construction, and diagnostic benchmarking of six reasoning LLMs using the MedQA multiple-choice dataset. Diagnostic accuracy, response latency, and connection reliability were evaluated. The section ends with a description of the statistical tests used to compare model performance and determine significance.

### 2.1 Dataset

MedQA (Medical Question Answering Dataset) [11] is a specialized benchmark designed to evaluate AI models on medically demanding question-answering tasks that approximate real examination-style clinical reasoning. The dataset is compiled from medical licensing and board examination questions from the United States, Mainland China, and Taiwan, where physicians are tested on professional knowledge and clinical decision-making ability. These questions span diverse medical domains and subspecialties, typically requiring a solid understanding of medical concepts grounded in textbook-based knowledge. This makes MedQA an essential tool for developing AI models capable of accurately addressing complex medical queries [3].

For the present study, we evaluated all models on the first 1,000 multiple-choice questions extracted from MedQA, with each question containing four answer options. This subset was selected as a pragmatic benchmarking sample to balance statistical stability with the practical constraints of repeated API-based evaluation across multiple models, including cost, rate limits, runtime, and logging consistency. A sample of this size remained sufficiently large to support comparative analyses of accuracy, latency, and connection reliability, while maintaining a feasible and reproducible workflow under uniform testing conditions. Importantly, the selected subset included questions originating from the different regional sources represented in MedQA and preserved broad diversity in medical subspecialties and question characteristics. In addition, medical textbooks provided within the MedQA resource were used to construct the local knowledge base for the RAG experiments.

### 2.2 Models

#### 2.2.1 ChatGPT o3-mini-high

ChatGPT o3-mini [12] is the first small reasoning model launched by OpenAI, supporting highly requested developer features, including function calling, structured outputs, and developer messages. Users can choose between three reasoning effort options–low, medium, and high–to optimize for their specific use cases. This study employed the ChatGPT o3-mini-high model, utilizing its API to generate responses to the questions.

#### 2.2.2 DeepSeek-R1

DeepSeek-R1 is a reinforcement learning-based LLM developed to enhance reasoning capabilities without relying on supervised fine-tuning (SFT). It achieves impressive results in various benchmarks, including math, coding tasks, and knowledge-based queries, while maintaining competitive performance across a range of tasks. We utilized its API to generate responses to the questions.

#### 2.2.3 DeepSeek-R1:8b

DeepSeek team has demonstrated that the reasoning patterns of larger models can be distilled into smaller models, resulting in better performance compared to the reasoning patterns discovered through reinforcement learning on small models [5].

Larger models demand greater GPU memory and storage resources. Considering the hardware costs affordable for small clinics, this study selected DeepSeek-R1-Distill-Llama-8B [13] (hereafter referred to as DeepSeek-R1:8b) for local deployment to generate responses to the questions. The model employs 4-bit quantization using the K_M method and contains approximately 8.03 billion parameters.

### 2.3 Tools

#### 2.3.1 Ollama

Ollama is an open-source LLM service tool designed to enable users to efficiently run models locally. It features a streamlined command-line interface and server, allowing users to easily download, run, and manage various open-source LLMs. In this study, Ollama was utilized to deploy the local model on a Windows PC.

#### 2.3.2 Chatbox AI

Chatbox AI is an AI client application and intelligent assistant, compatible with various advanced AI models and APIs. When integrated with Ollama, it provides a streamlined and user-friendly front-end UI. In this study, it was employed for debugging the locally deployed model.

#### 2.3.3 RAGFlow

RAGFlow is an open-source Retrieval-Augmented Generation(RAG) engine based on deep document understanding. It offers a streamlined RAG workflow for businesses of any scale, combining LLMs to provide truthful question-answering capabilities, backed by well-founded citations from various complex formatted data. In this study, it was used to construct the local knowledge base.

#### 2.3.4 Nomic-embed-text

Nomic-embed-text [14] is a large context-length text encoder that transforms text into fixed-length vectors, called embeddings, which capture the semantic relationships between texts. This allows semantically similar texts to have similar vector representations. These embeddings are crucial for tasks like text classification, sentiment analysis, and information retrieval, helping models better understand and process language. In this study, it was utilized to parse medical textbooks.

### 2.4 Hardware environment

The performance and responsiveness of a locally deployed model are directly influenced by the computing hardware, particularly the GPU capabilities. Deploying a model such as DeepSeek-R1:671B is estimated to require at least 350GB of VRAM for balanced performance, along with considerations for stable power supply and effective cooling-making it a feasible option only for large hospitals or enterprises. Table 1 outlined the hardware specifications used in this study for local model deployment. A laptop GPU with 8 GB VRAM was chosen to approximate the hardware capacity available in most small-to-medium clinical settings, where mobile and offline inference solutions are frequently used. This configuration was not intended to demonstrate maximal throughput. Instead, it was designed to represent common feasibility limits encountered in routine clinical practice. Deployment trade-offs were assessed based on these realistic hardware constraints.

**Table 1:** Computer hardware specification in this study.

Hardware component	Specifications
Central processing unit	Intel Core i7 processor 14650HX
Graphics processing unit	NVIDIA GeForce RTX 4060 Laptop GPU (8 GB)
Random access memory	32 GB DDR5 5600 MHz (16 GB + 16 GB)
Solid state drive	KNIGSTON OM8PGP41024N-A0 1024 GB
	Predator SSD GM7 1024 GB

In this study, Ollama was deployed on a computer to integrate the DeepSeek-R1:8b and Nomic-embed-text into the local environment. In settings such as hospitals, where internet access may be restricted or limited to internal networks, GGUF files can be transferred via USB drives or file transfer software for offline installation.

RAGFlow was utilized to integrate DeepSeek-R1, ChatGPT o3-mini-high, and DeepSeek-R1:8b as chat models, whereas Nomic-embed-text served as the embedding model. A knowledge base comprising 18 English medical textbooks was constructed in RAGFlow. These textbooks were drawn from the MedQA textbook collection and covered core disciplines including anatomy, biochemistry, cell biology, pathology, pharmacology, physiology, internal medicine, neurology, obstetrics and gynecology, pediatrics, psychiatry, and surgery. The books were not arbitrarily selected by us; rather, they correspond to the document collection assembled by the original MedQA authors. According to the MedQA dataset paper, this document collection was built from medical textbooks “widely used by medical students and USMLE takers,” and the same English textbook collection was used for both the USMLE and TWMLE subsets [11]. Thus, our knowledge base was intended to provide exam-relevant background knowledge likely to contain evidence useful for answering MedQA items, because it followed the textbook corpus originally assembled for this task rather than an independently expanded corpus with different scope and curation principles. The knowledge base was ingested with default chunking: documents were split into segments of roughly 256 tokens each, using RAGFlow’s “naive” parser. Each chunk was embedded as a vector using the nomic-embed-text model. These chunk embeddings were indexed for semantic similarity search. On the query side, RAGFlow’s retrieval component used a hybrid similarity metric, combining embedding cosine similarity with keyword overlap. A similarity threshold of 0.3 was applied to filter low-relevance chunks, and retrieval scoring was primarily weighted toward lexical similarity (keyword similarity weight = 0.7, vector similarity weight = 0.3). For each user question, the system retrieved the top 7 most similar chunks (Top N = 7) to serve as context for the LLM. No rerank model was applied. To ensure reproducibility and fairness, temperature (0.1) and output token limits were the only decoding parameters explicitly aligned across all models, while all other sampling, penalty, and output-length parameters were left at their vendor-defined default settings, as they were not consistently exposed or guaranteed to take effect across endpoints. No multi-turn conversational memory or knowledge-graph-based retrieval was employed, so each query was answered independently using the retrieved textual evidence. Diagnostic retrieval evaluation was conducted prior to answer generation to verify whether answer-bearing chunks were successfully recalled. To isolate the impact of retrieval quality, we performed RAG ablation by comparing model accuracy with and without retrieved context. In the ablation setup, models were provided with only the highest-ranked chunk to evaluate the sufficiency of the most relevant retrieved information. Retriever performance is quantified using first-pass passage-level precision and recall on a 50-question validation subset, yielding a mean precision of 0.82 and recall of 0.88.

Python code was implemented to achieve the following functionality:

1. Model integration—invoking the APIs of DeepSeek-R1 and ChatGPT o3-mini-high, and querying the locally deployed DeepSeek-R1:8b via Ollama. RAGFlow was used as a retrieval and orchestration engine that exposes a local API endpoint for the Python code to submit questions and receive the final responses.
2. Response retrieval—the system prompt was set as follows: “*You are a medical expert. Please select the correct option from the four choices A, B, C, or D. Do not include any explanation, option content, or additional information. Only return the letter corresponding to the correct option (A, B, C, or D).*” The temperature was configured to 0.1, whereas other parameters such as Max Tokens, Top-p, and Presence penalty were not consistently exposed across endpoints and therefore remained at vendor defaults.
  Several prompt variants were tested during pilot runs. Prompt refinement aimed primarily to improve output standardization for automated parsing rather than to optimize benchmark accuracy. The final prompt was chosen because it produced the most workable format for downstream analysis, although some models still generated explanations or other noncompliant output. The same prompt framework was used for all models.
  The question stem and answer options for each selected MedQA item were combined with the standardized prompt and then sequentially submitted to the six AI models. For each query, a complete log was generated, including the full response text produced by the model—covering both the reasoning process and the final answer output—as well as the corresponding response time. These logs were subsequently used for answer extraction, response-format assessment, and downstream statistical analysis.
3. Delay insertion—a 1 s interval was introduced between each query to prevent quota problems caused by frequent requests.
4. Timeout detection and connection failure definition—If a model failed to establish a valid connection on the first request attempt, exceeded 120 s without returning a complete response, or returned an invalid answer, it was classified as a connection failure. For each failure, we also recorded the start timestamp of the initial request and the elapsed time until the failure was triggered (timeout or response error). This elapsed time was stored as the response time for the failed first attempt, reflecting the real latency impact of connection timeout events. Subsequent retries were performed only to obtain a complete answer for accuracy evaluation and were excluded from both response time and connection failure rate statistics.
5. Result aggregation—the selected answer was extracted from the final answer portion of each log and compared against the correct answer to determine accuracy. A response was considered automatically scorable when the model’s output adhered to the required format, enabling the extraction script to reliably identify a single answer letter (A, B, C, or D).
  However, some responses did not strictly follow the required format and therefore underwent manual review for final verification and scoring. Manual adjudication was performed independently by two reviewers, and ambiguous cases were resolved by discussion until consensus was reached. The same scoring rule was applied uniformly across all models. Responses containing multiple answer choices were scored as incorrect. Responses stating that none of the provided options was correct were likewise scored as incorrect. Responses that contained only reasoning or explanatory text without a single explicit final answer choice were also scored as incorrect. When a response included additional irrelevant text but still yielded a single identifiable answer choice, that answer was used for scoring. This procedure was adopted to ensure consistent treatment of noncompliant outputs across models.
  First-attempt response time was recorded for all queries, including failures. When the first attempt failed, the elapsed time from the initial request start to the failure trigger was recorded as the response time for that failure event. This approach ensured that latency distributions included the real-time cost of connection timeout incidents. Retries were executed only to obtain a completed answer for correctness comparison and were not used to replace or overwrite the first-attempt latency or connectivity metrics.

### 2.5 Statistical analysis

All analyses were conducted on 1,000 problem-solving instances using SPSS 29.0 and Python 3.12.3 (statsmodels). Non-parametric methods were employed due to non-normal distributions of the data (Kolmogorov-Smirnov test: p<0.01 for all response time variables). Statistical significance was defined as p<0.05, with Bonferroni correction applied for multiple comparisons where appropriate.

For accuracy and connection failures (binary outcomes), differences across six models were evaluated using Cochran’s Q tests, followed by pairwise McNemar tests with Bonferroni correction (adjusted significance threshold: α = 0.0033). Inter-rater agreement for multi-model comparisons was quantified using Fleiss’ kappa, whereas pairwise consistency was assessed with Cohen’s kappa (both incorporating Bonferroni correction). Response times were analyzed using Friedman tests to identify global differences, with post hoc pairwise contrasts examined via Wilcoxon signed-rank tests (Bonferroni-adjusted).

Effect sizes were interpreted as follows: ϕ (phi coefficient) thresholds of 0.10 (weak), 0.30 (moderate), and >0.50 (strong); κ (kappa) values of <0.20 (slight), 0.21–0.40 (fair), and 0.41–0.60 (moderate agreement).

## 3 Results

For clarity, in the following text, the six models—ChatGPT o3-mini-high, DeepSeek-R1, and DeepSeek-R1:8b, along with their knowledge base-integrated versions—were referred to as: O3, R1, R1:8b, O3-kb, R1-kb and 8b-kb. However, in figures and tables, the full model names were retained for better readability.

### 3.1 Accuracy performance

**Figure 1:**
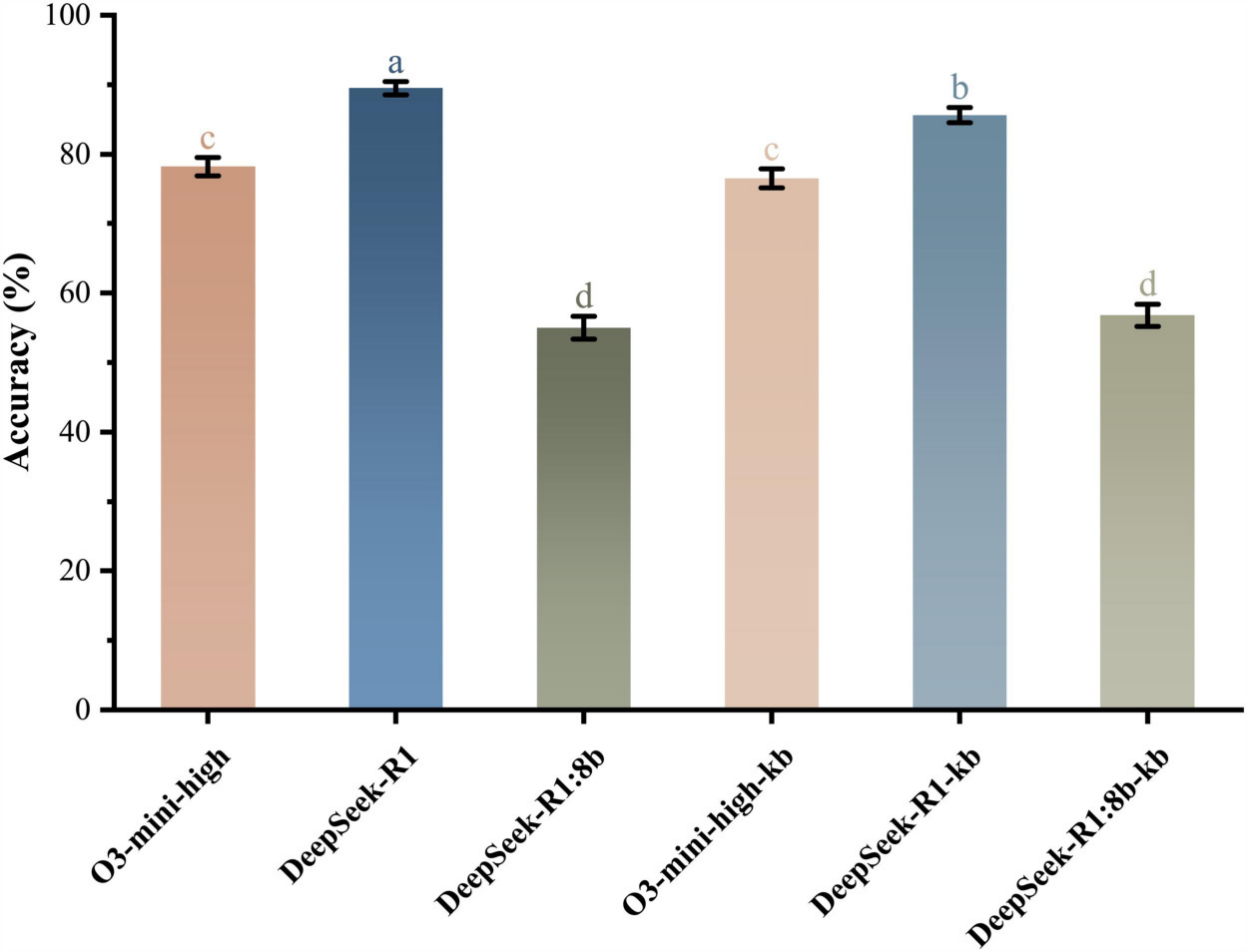
Accuracy comparison of six models. Vertical error bars indicate standard errors of proportions. Models sharing the same lowercase letter(s) belong to homogeneous subsets without statistically significant differences.

Accuracy analysis demonstrated significant performance stratification across models (Cochran’s Q(5)=741.965, p<0.001, ϕ=0.385 [moderate effect]), as illustrated in Figure 1. The highest accuracy was observed in R1 (89.5%, 95% CI: 87.4%–91.2%) and R1-kb (85.6%, 95% CI: 83.2%–87.7%), whereas the lowest performance occurred in R1:8b (55.0%, 95% CI: 51.9%–58.5%) and R1:8b-kb (56.8%, 95% CI: 53.8%–59.9%). Knowledge base (KB) integration exhibited model-dependent effects: R1-kb showed a statistically significant 4.36% accuracy reduction compared to R1 (raw p=0.0002), whereas O3-kb demonstrated a nonsignificant 2.2% decrease relative to O3 (raw p=0.183).

The consistency analysis revealed fair inter-model agreement across all models (Fleiss’ κ=0.269, p<0.05), with statistically significant but predominantly poor pairwise consistency. The highest agreement was observed between the R1 and R1-kb models (κ=0.59), highlighting their operational similarity, whereas the majority of comparisons demonstrated insufficient alignment despite meeting statistical significance thresholds.

### 3.2 Response time efficiency

**Figure 2:**
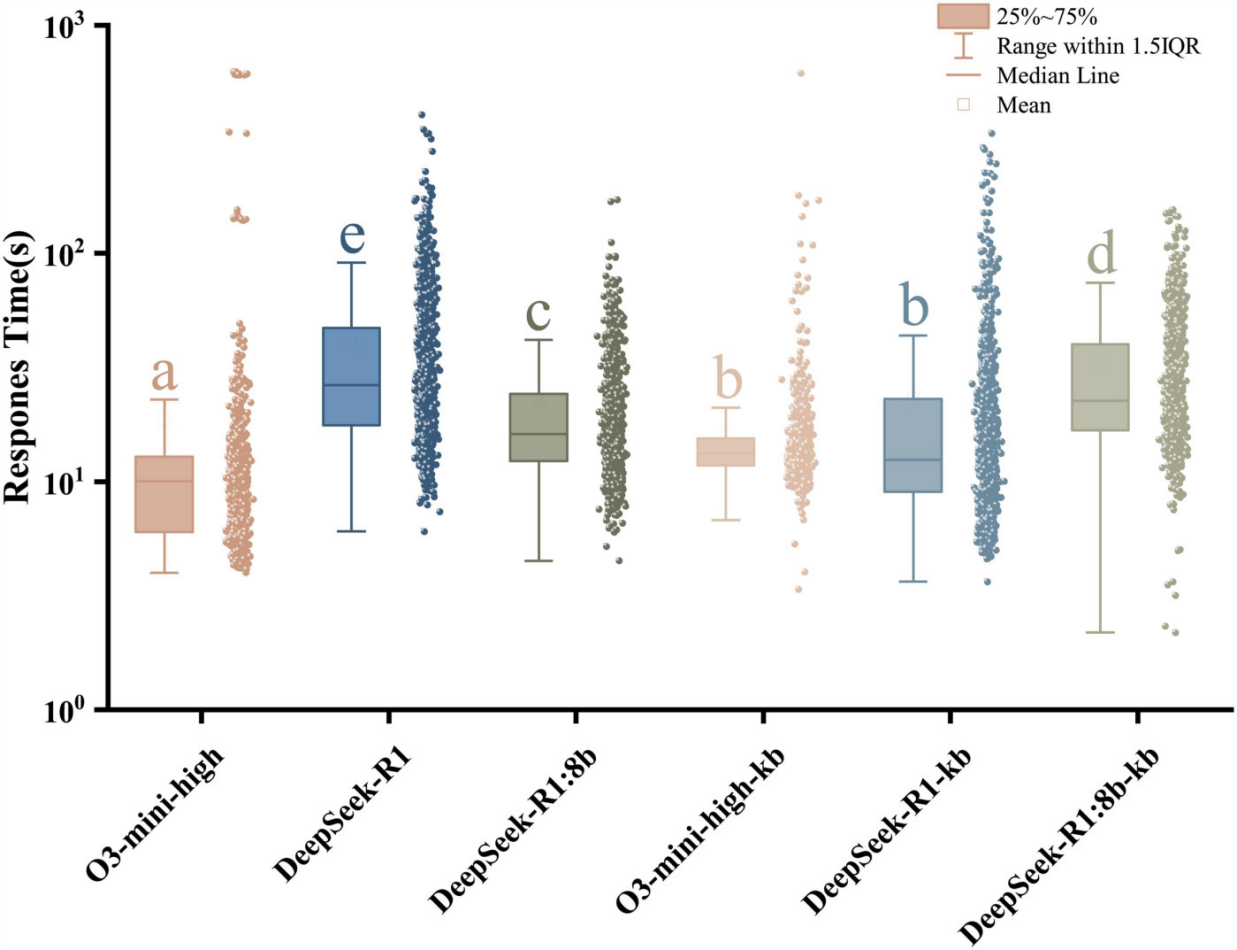
Boxplot of response time ($\log_{10}$ transformed). Boxes span the interquartile range (IQR), central line represents the median, whiskers extend to 1.5×IQR. Models sharing the same lowercase letter(s) belong to homogeneous subsets without statistically significant differences.

The response times of each model were presented in Figure 2. To reduce the impact of extreme values and better visualize the distribution of response times, a logarithmic transformation ($\log_{10}$) was applied for visualization only, while all statistical tests were conducted on the raw response times. Friedman test confirmed response time differences: $\chi^{2}(5)=1,860.229$, p<0.001. All pairwise comparisons except O3-kb vs. R1-kb (raw p=0.0087>0.0033) demonstrated statistically significant differences (all raw p<0.0001). The O3 series models exhibited shorter overall response times, whereas the R1 model demonstrated the longest latency. Relative to R1, R1-kb reduced response times by 52.9%. The locally deployed model (R1:8b) showed 39.1% faster responses than cloud-based R1.

In addition to median response time and interquartile range, we summarized tail latency using the 95th percentile (P95) response time, defined as the response time below which 95% of requests were completed, across the 1,000 questions for each model. The P95 response times were 26.81 s (O3), 111.49 s (R1), 50.87 s (R1:8b), 26.01 s (O3-kb), 73.97 s (R1-kb), and 77.45 s (8b-kb).

### 3.3 Connection reliability

**Figure 3:**
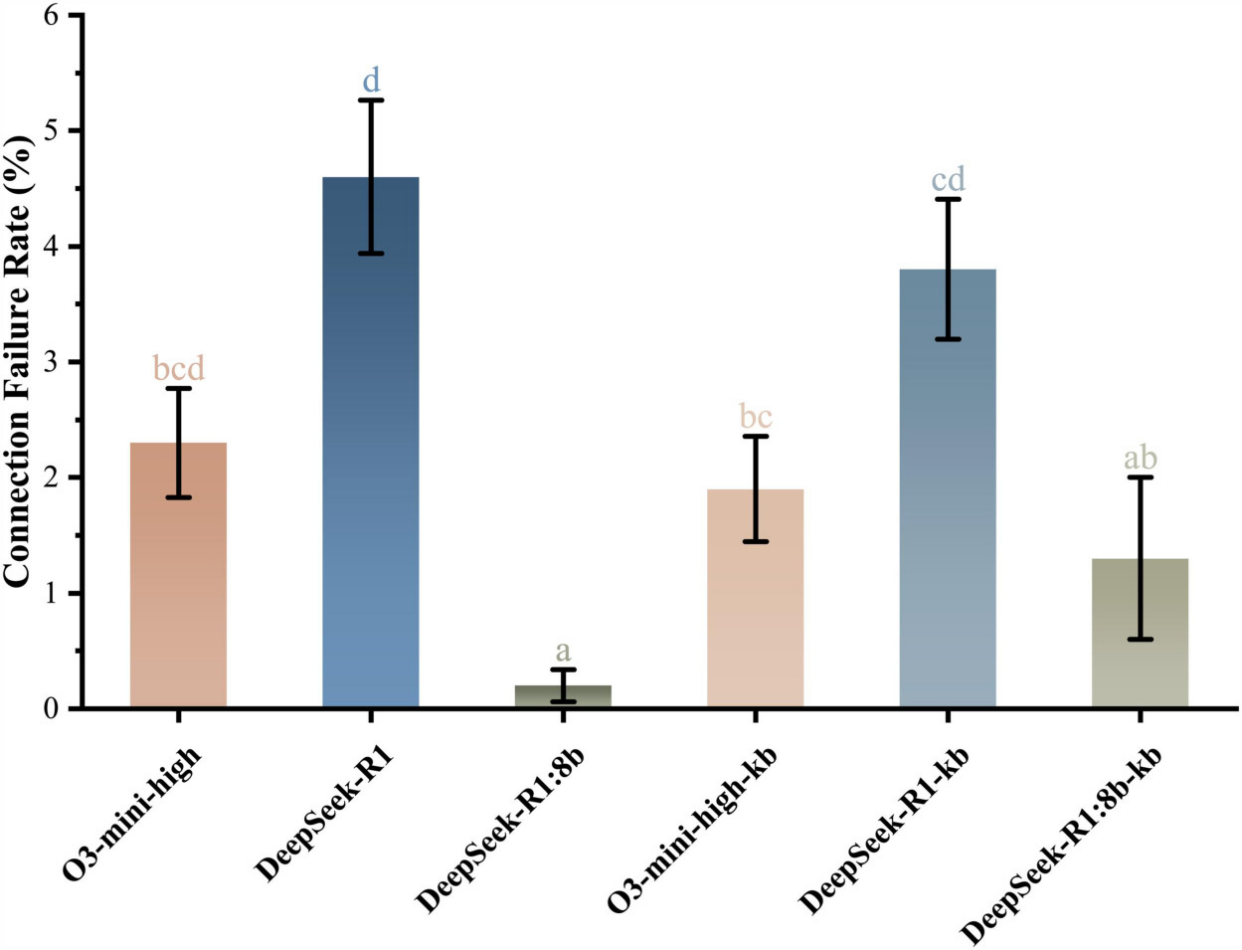
Comparison of connection failure rates among six models. Vertical bars denote standard errors of proportions. Models sharing the same lowercase letter(s) belong to homogeneous subsets without statistically significant differences.

Connection reliability analysis demonstrated significant deployment-mode dependency (Cochran’s Q(5)=56.525, p<0.001, ϕ=0.106 [weak effect]), as illustrated in Figure 3. Local deployments exhibited superior connection reliability, with R1:8b achieving 0.2% failure rate (95% CI: 0.0%–0.5%) and its knowledge-enhanced variant R1:8b-kb at 1.3% (95% CI: 0.7%–2.0%). In contrast, cloud-based implementations showed substantially higher failure rates: R1 at 4.6% (95% CI: 3.3%–6.0%) and R1-kb at 3.8% (95% CI: 2.5%–5.0%). The locally deployed R1:8b model demonstrated a 95.7% relative risk reduction in failure rate compared to the cloud-based R1.

As summarized in Table 2, R1 achieved the highest accuracy (89.5%, 95% CI 87.4–91.2) but exhibited higher connection failure rates (4.6%, 95% CI 3.3–6.0), whereas R1:8b models demonstrated enhanced connection reliability (failure rate 0.2%, 95% CI 0.0–0.5) with comparatively lower accuracy (55.0%, 95% CI 51.9–58.5).

**Table 2:** Performance metrics with composite significance markers.

Model	Accuracy(%) Proportion [95% CI]	Response time (s) Median [IQR]	Connection failure (%) Proportion [95% CI]
Test statistic	Q(5)=741.965, p<0.001	$\chi^{2}(5)=1,860.229$, p<0.001	Q(5)=56.525, p<0.001
O3-mini-high	78.2 [75.5, 80.7]c	10.05 [6.00–12.87]a	2.3 [1.4, 3.3]bcd
DeepSeek-R1	89.5 [87.4, 91.2]a	26.54 [17.67–47.32]e	4.6 [3.3, 6.0]d
DeepSeek-R1:8b	55.0 [51.9, 58.5]d	16.17 [12.26–24.24]c	0.2 [0.0, 0.5]a
O3-mini-high-kb	76.5 [73.9, 79.2]c	13.30 [11.79–15.49]b	1.9 [1.1, 2.9]bc
DeepSeek-R1-kb	85.6 [83.2, 87.7]b	12.49 [8.99–23.11]b	3.8 [2.5, 5.0]cd
DeepSeek-R1:8b-kb	56.8 [53.8, 59.9]d	22.61 [16.74–39.91]d	1.3 [0.7, 2.0]ab

Groups with the same letter are not significantly different (Bonferroni-corrected p>0.0033). Letter comparisons are column-specific.

## 4 Discussion

### 4.1 Overall performance trade-offs across reasoning models

This study benchmarked three reasoning LLM configurations within a standardized MedQA framework, demonstrating that model performance is best understood as a multifaceted trade-off involving accuracy, latency, output compliance, and connection reliability, rather than through accuracy alone. DeepSeek-R1 achieved the highest diagnostic accuracy in our evaluation, outperforming ChatGPT o3-mini-high, though this advantage was accompanied by substantially longer response times. The relatively low inter-model agreement, as measured by Cohen’s κ, suggests that these systems may rely on distinct internal reasoning strategies, training corpora, or alignment objectives, even when responding to identical multiple-choice medical questions. Furthermore, certain models—particularly those in the DeepSeek-R1:8b series—exhibited weaker adherence to the required output format, increasing the need for manual review and underscoring the importance of structured-output control in downstream clinical deployment.

These findings are directionally consistent with prior work demonstrating that stronger reasoning or medically adapted models can achieve high performance on medical QA tasks. Singhal et al. reported that Med-PaLM 2 achieved up to 86.5% accuracy on MedQA following domain-adapted training and improved grounding strategies, illustrating that expert-level performance is attainable when both model and evaluation pipeline are tightly optimized [3]. Chen et al. further showed that model performance declines on more challenging medical QA benchmarks requiring deeper case comprehension and explanation quality, suggesting that apparent reasoning performance depends substantially on dataset design and evaluation scope [4]. Our findings align with this broader pattern: reasoning models can perform strongly on exam-style medical QA, yet observed performance remains sensitive to architecture, prompting, and benchmark conditions. We therefore provide a narrative comparison to situate our findings within the broader literature.

From a response-time perspective, ChatGPT o3-mini-high demonstrated the most agile tail performance (P95: 26.81 s), whereas DeepSeek-R1 incurred substantially higher tail latency (P95: 111.49 s), revealing a meaningful worst-case waiting-time burden despite its superior accuracy. Additionally, both DeepSeek-R1 and ChatGPT o3-mini-high occasionally experienced connection timeouts or produced incomplete outputs, contributing to higher first-attempt failure rates in cloud-based configurations. While prior medical QA studies have largely emphasized answer correctness and reasoning quality [3, 4], our work introduces a broader evaluation lens by incorporating deployment-oriented metrics such as P95 latency and first-attempt connection reliability. This expanded perspective complements existing benchmark literature by demonstrating that strong reasoning performance does not automatically confer operational suitability under realistic usage constraints.

### 4.2 Effects and limitations of knowledge-base augmentation

Retrieval-augmented generation (RAG) has been widely proposed as a method to enhance factual grounding in medical question answering. Prior work has demonstrated that medical RAG can substantially improve QA performance when the retrieval corpus, retriever, and generator are carefully aligned. For instance, Xiong et al. reported that MedRAG improved the accuracy of six different LLMs by up to 18% over chain-of-thought prompting across multiple medical QA datasets [9]. Similarly, Sohn et al. showed that rationale-guided retrieval could improve performance by up to 6.1% across three medical question-answering benchmarks, underscoring the importance of rationale-based querying, distractor filtering, and balanced retrieval across biomedical corpora [10]. Collectively, these studies suggest that RAG effectiveness is highly contingent on retrieval design, corpus quality, and architectural compatibility, rather than being uniformly beneficial across all settings.

In our benchmark, however, knowledge-base integration did not universally improve performance. DeepSeek-R1 exhibited a 4.36% decline in accuracy with RAG, while o3-mini-high and DeepSeek-R1:8b showed no statistically significant improvement. This divergence from prior findings is informative rather than contradictory, and several factors may explain it. First, our knowledge base was limited to the 18-textbook corpus distributed with MedQA. While directly relevant to the examination domain, this corpus is not equivalent to a comprehensive clinical knowledge environment; it lacked specialized resources such as subspecialty reference texts, structured drug databases, or up-to-date clinical guidelines. In contrast, prior RAG studies were conducted under broader and more carefully engineered retrieval settings, including multi-corpus biomedical resources and stronger retrieval filtering strategies [9, 10]. Although these studies were not directly designed around the same MedQA textbook collection, their retrieval pipelines were more extensively optimized than ours, which may partly explain the difference in observed performance gains. Second, our implementation did not include a reranking stage, as incorporating a reranking model would have increased system response time under our experimental constraints. Nevertheless, prior studies suggest that reranking and relevance filtering can improve retrieval precision and downstream generator accuracy by prioritizing answer-bearing evidence over merely topically related chunks [15]. Third, retrieval may interact differently with distinct model architectures [16]. If a model is not explicitly optimized to incorporate retrieved context, the added information may compete with its internal parametric knowledge rather than reinforce it. Fourth, retrieval introduces additional context and processing overhead, which may dilute reasoning capacity—particularly in smaller models. At the same time, differences observed under the RAG setting should not be attributed solely to the underlying LLMs. They may also reflect characteristics of the specific RAG pipeline used in this study, including chunking strategy, embedding model, retrieval depth, prompt construction, context handling, and the absence of a reranking stage. Although limited retrieval-level analyses were performed, the present study did not include a systematic component-wise ablation of the RAG pipeline. Therefore, we could not quantify the relative contribution of the underlying LLMs vs. specific retrieval design choices to the observed performance differences. Accordingly, the RAG-related findings should be interpreted as reflecting the interaction between each model and the specific pipeline implemented here, rather than as a pure measure of intrinsic model capability.

Taken together, our results support a more nuanced perspective on medical RAG. They do not imply that RAG is ineffective in clinical contexts; rather, they demonstrate that its benefits are not universally additive and remain contingent on multiple factors—including corpus scope, retriever quality, reranking strategy, context filtering, and model-level compatibility. This interpretation aligns closely with prior work by Sohn et al. and Xiong et al., both of which underscore that retrieval quality and system design are central determinants of medical RAG performance [9, 10].

### 4.3 Deployment-oriented implications under practical constraints

Beyond accuracy, our findings demonstrate that real-time clinical utility involves a fundamental trade-off among reasoning capability, latency, and connection reliability. In our benchmark, o3-mini-high exhibited the most agile tail performance (P95: 26.81 s). Conversely, DeepSeek-R1 incurred substantially higher tail latency (P95: 111.49 s), revealing a significant worst-case waiting-time burden despite its superior reasoning accuracy. This latency penalty was partially offset by knowledge-base integration, which reduced DeepSeek-R1’s P95 latency to 73.98 s.

Connection reliability analysis further revealed significant deployment-mode dependency. The locally deployed DeepSeek-R1:8b model exhibited a near-zero failure rate of 0.2% (95% CI: 0.0%–0.5%), representing a 95.7% relative risk reduction compared with its cloud-based counterpart (4.6%, 95% CI: 3.3%–6.0%). However, this connection reliability advantage did not extend to reasoning performance: under personal-computer-level hardware constraints, DeepSeek-R1:8b achieved lower accuracy than the cloud-based DeepSeek-R1 model. Thus, local deployment should be understood not as an unconditional advantage, but rather as a context-dependent trade-off between operational continuity and model capability.

These findings point toward differentiated deployment roles. High-reasoning cloud models may be better suited for non-urgent or asynchronous tasks—such as complex case review or second-opinion support—where answer quality takes precedence over immediate responsiveness. In contrast, locally deployed configurations may be preferable in environments where connectivity is unstable or where uninterrupted access is operationally critical. More broadly, the emergence of open-weight reasoning models like DeepSeek-R1 and its distilled variants has made practical deployment considerations more salient, especially in resource-constrained settings [5]. Importantly, these implications should be interpreted as deployment-relevant benchmarking evidence derived from a controlled MedQA setting, rather than as direct proof of real-world clinical effectiveness.

### 4.4 Limitations and future directions

Several limitations should be acknowledged. First, this study evaluated models on a fixed subset of 1,000 multiple-choice questions drawn from the MedQA dataset. While this sample was sufficiently large for comparative benchmarking, it was not designed as a stratified random sample and reflects an examination-style task rather than the full complexity of real-world clinical workflows. Prior work has demonstrated that model performance can vary substantially on more challenging clinical-case benchmarks, particularly on tasks requiring explanation quality over answer selection alone [4].

Second, while we incorporated latency and connection reliability, several additional properties critical for downstream deployment were not systematically evaluated—including safety alignment, hallucination rates, explanation quality, and instruction-following robustness under adversarial or ambiguous prompting. These dimensions represent important directions for future work.

Third, the RAG knowledge base was limited to the textbook corpus distributed with MedQA and therefore did not constitute a comprehensive clinical knowledge environment. It lacked specialty-specific textbooks, updated clinical guidelines, consensus statements, and structured pharmacologic reference resources. This may partly explain the inconsistent RAG effects observed in our benchmark. Future work should investigate whether broader and more clinically targeted corpora—together with reranking, improved retrieval–architecture alignment, and systematic component-wise ablation of the RAG pipeline—can yield more robust and consistent gains.

Finally, this study is fundamentally a benchmarking exercise rather than a clinical validation study. Accordingly, the findings should be interpreted as evidence of comparative technical performance under standardized conditions, not as direct support for real-world clinical deployment. Future research should extend beyond exam-style question answering toward prospective, workflow-integrated, and clinically validated evaluations of reasoning models.

In summary, this study provides deployment-relevant benchmarking evidence characterizing the trade-offs among reasoning performance, latency, connection reliability, and deployment mode within a standardized MedQA evaluation framework. Rather than offering direct clinical deployment guidance, it helps clarify which technical trade-offs warrant further investigation in future healthcare-oriented evaluations of reasoning models.

## 5 Conclusion

This study benchmarked reasoning LLMs under a standardized MedQA setting and showed that model performance is best understood as a trade-off among accuracy, latency, connection reliability, and deployment mode. DeepSeek-R1 achieved the highest accuracy, ChatGPT o3-mini-high showed the lowest tail latency, and locally deployed DeepSeek-R1:8b exhibited the strongest connection reliability. Knowledge-base augmentation did not universally improve performance, indicating that RAG effectiveness depends on corpus quality, retrieval design, and model–system compatibility. These findings provide deployment-relevant benchmarking evidence for future healthcare-oriented AI evaluation, but they should not be interpreted as direct validation for real-world clinical deployment. Future studies should assess reasoning models using richer knowledge resources and more realistic clinical workflows.

## Data Availability

Publicly available datasets were analyzed in this study. This data can be found here: https://github.com/jind11/MedQA.
